# The Notch signaling-regulated angiogenesis in rheumatoid arthritis: pathogenic mechanisms and therapeutic potentials

**DOI:** 10.3389/fimmu.2023.1272133

**Published:** 2023-10-26

**Authors:** Fang Zhao, Yini He, Zhihao Zhao, Jiarong He, Hong Huang, Kelong Ai, Liang Liu, Xiong Cai

**Affiliations:** ^1^ Department of Rheumatology of The First Hospital and Institute of Innovation and Applied Research in Chinese Medicine, Hunan University of Chinese Medicine, Changsha, Hunan, China; ^2^ Xiangya School of Pharmaceutical Sciences, Central South University, Changsha, Hunan, China; ^3^ Institute (College) of Integrative Medicine, Dalian Medical University, Dalian, Liaoning, China; ^4^ Department of Neurosurgery, The Second Xiangya Hospital, Central South University, Changsha, Hunan, China; ^5^ State Key Laboratory of Dampness Syndrome of Chinese Medicine, The 2nd Affiliated Hospital of Guangzhou University of Chinese Medicine, Guangzhou, Guangdong, China

**Keywords:** rheumatoid arthritis, angiogenesis, Notch signaling, adipokines, epigenetic regulation

## Abstract

Angiogenesis plays a key role in the pathological process of inflammation and invasion of the synovium, and primarily drives the progression of rheumatoid arthritis (RA). Recent studies have demonstrated that the Notch signaling may represent a new therapeutic target of RA. Although the Notch signaling has been implicated in the M1 polarization of macrophages and the differentiation of lymphocytes, little is known about its role in angiogenesis in RA. In this review, we discourse the unique roles of stromal cells and adipokines in the angiogenic progression of RA, and investigate how epigenetic regulation of the Notch signaling influences angiogenesis in RA. We also discuss the interaction of the Notch-HIF signaling in RA’s angiogenesis and the potential strategies targeting the Notch signaling to improve the treatment outcomes of RA. Taken together, we further suggest new insights into future research regarding the challenges in the therapeutic strategies of RA.

## 1 Introduction

Rheumatoid arthritis (RA) is a prototypic autoimmune disease characterized by progressive inflammatory synovitis. Severe RA is accompanied by clinical sequelae and has a 50% risk of cardiovascular mortality. In global epidemiology, rheumatoid arthritis has a very high global age-standardized prevalence (7.4% of the total population) and incidence (8.2% of the total population) (1). In the pathogenesis of RA, inflammation is the background response that drives resident interstitial synovial cells to acquire a pro-angiogenic phenotype, resulting in dramatic changes in synovial structure and function (2). Angiogenesis plays an early and key role in the process of synovitis. In the inflammatory environment, vessels firstly undergo a period of high inflammation, followed by a period of intense increase in blood vessel growth. Newly formed blood vessels provide oxygen and nutrition for proliferative synovitis cells, further promoting the progression of synovitis and promoting joint invasion and destruction. Angiogenesis is essential for supplying nutrients and oxygen to the proliferative joints of patients with RA, relying on an extremely complex network system consisting of highly interactive and mutually promoted extracellular matrix and stromal cells (3).

Angiogenesis is the process of the formation of new capillaries that develop from preexisting vessels. Angiogenesis is critical for many physiological processes, including embryonic organ development, tissue repair, remodeling, and wound repair (4), and it initiates when vascular endothelial growth factor (VEGF) and fibroblast growth factor (FGF) bind to several cognate receptors on endothelial cells (ECs). Activated ECs induce the disassembly of tight junctions, leading to an increase in vascular permeability. Subsequently, matrix metalloproteinases (MMPs) degrade the vascular basement membrane, accelerating endothelial migration, proliferation, and the stability of new tubes. Interconnected tubules accelerate the construction of vessel networks, which are regulated by Ang1, a pro-angiogenic factor, to potentiate pericyte recruitment and facilitate blood flow (5).

Notch signaling is a highly conserved intercellular signaling mechanism that directs cell fate specification and proliferation and controls cell differentiation and processes in both embryonic and adult tissues (6). Notch signaling is a pivotal regulator of vessel formation and is involved in EC-pericyte interactions and the coupling of osteogenesis and angiogenesis (7). Notch signaling mainly consists of four parts: ligands, receptors, DNA-binding proteins, and downstream gene transcription (8). The epigenetic regulation of Notch signaling determines the specificity of Notch target genes in different cell types (9). As for the effect of the epigenetic regulation of Notch signaling in angiogenesis, it is worth further investigation.

Many human diseases, including rheumatoid arthritis, are associated with aberrant Notch signaling (10). Several studies have shown that the Notch signaling pathway is critical for hypoxia-induced proliferation and angiogenesis (11). Under hypoxic conditions, the interaction between Notch-1 and hypoxia-inducible factor (HIF)-1α regulates invasion and angiogenesis in the progression of inflammatory arthritis (12). Notably, no review article has been published to date from the perspective of Notch signaling regulation of angiogenesis in RA.

Therefore, in this review, we have summarized the unique roles of stromal cells and adipokines and their influence on the progression of RA angiogenesis. A comprehensive description of Notch signaling and its epigenetic regulation is provided. We describe the role of the epigenetic regulation of Notch signaling in stromal cells and adipokines during angiogenesis. Then, we further elucidate the interaction of HIF-Notch signaling in angiogenesis. Finally, we discuss the potential strategies to target Notch signaling for the treatment of RA. Based on our discussion, we suggest new insights for future research regarding the challenges of developing novel treatment strategies for RA.

## 2 Angiogenesis in rheumatoid arthritis

**Figure 1:**
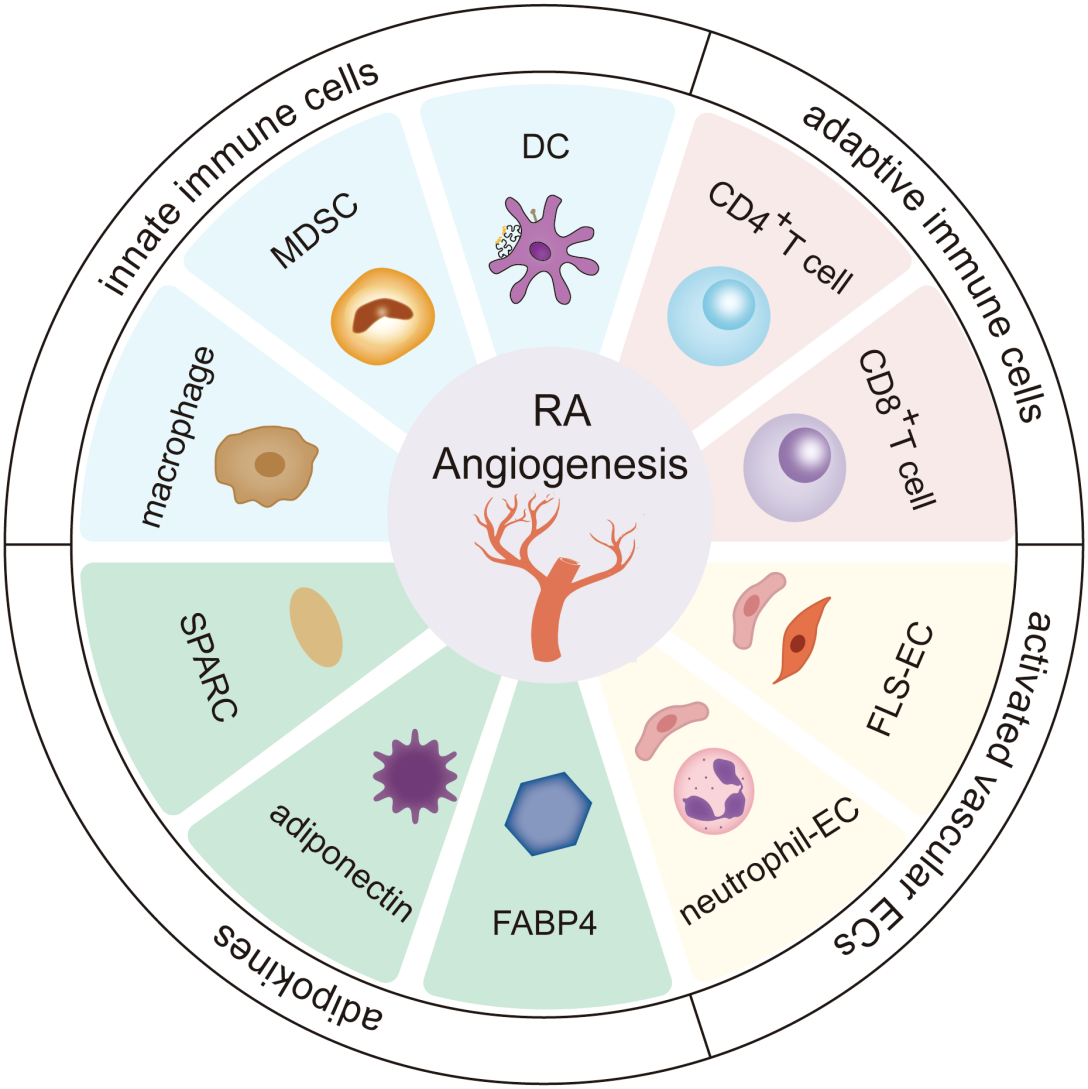
The role of stromal cells and adipokines in RA angiogenesis. Innate immune cells (including dendritic cells, cells-Myeloid-derived suppressor cells, and macrophages), adaptive immune cells (including CD4^+^ T cells and CD8^+^ T cells), vascular endothelial cells, and adipokines (including fatty acid-binding protein 4 (FABP4), adiponectin, secreted protein acidic and rich in cysteine (SPARC)) greatly influence the progression of angiogenesis in the RA microenvironment. The cross-talk interactions of stromal cells and adipokines form a continuous cycle, further deteriorating RA.

Angiogenesis in rheumatoid arthritis is an intricate process that depends on an extremely complex network system involving the extracellular matrix and stromal cells, by which innate immune cells, adaptive immune cells, vascular endothelial cells, and adipokines can turn on the switch for vessel formation to enhance cartilage erosion and pannus formation (**Figure 1**).

### 2.1 Innate immune cells-dendritic cells

Dendritic cells (DCs), which connect the innate and adaptive immune systems, are heterogeneous cell populations essential for immunity and maintaining immune tolerance due to their specialized antigen-presenting capabilities (13). DCs also represent potential therapeutic targets for angiogenesis and lymphangiogenesis in chronic and tumor-associated inflammation (14).

The process involves the following actions: (I) DCs secrete and release angiogenic growth factors, including vascular endothelial growth factor-A (VEGF-A), FGF2, and endothelin-1 (ET-1). These factors have the properties of promoting angiogenesis by activating transcription factors, including CREB, HIF-1α, and STAT3 (15). (II) DCs release angiogenic mediators directly or indirectly by expressing pro-angiogenic chemokines. The upregulated CXCL8, CCL2, and CCL21 chemokines can directly induce angiogenesis by binding to ECs (16, 17). Whereas, the actions of CXCL1, CXCL2, CXCL3, CXCL5, and CXCL7 are indirect, occurring via recruitment of NK cells, neutrophils, monocytes, and macrophages, which in turn promote angiogenesis by producing pro-angiogenic transmitter substances that induce endothelial cell aggregation (18–20). (III) DCs may contribute to the activation of vasculogenic events through transdifferentiation into ECs upon exposure to VEGF (14).

Recent studies have found that the precursors of conventional dendritic cells (pre-cDCs), monocyte-derived DCs (mo-DCs), plasmacytoid DCs (pDCs), and Langerhans cells (LCs), which belong to the most studied specialized subtypes, are major players in the chemotaxis and migration of DCs during inflammation and immunity (21). Studies have indicated that an increased level of pre-DCs in the peripheral blood of RA patients with relatively poor prognoses is a key indicator of treatment resistance (22). Mo-DCs induce deleterious Th17 cell responses and participate in the chronic inflammatory microenvironment and bone loss in RA (23). ERK1/2 modulated mo-DCs may also contribute to regulating angiogenesis (24). pDCs are a major link between anomalous immunity and angiogenesis in rosacea (25). LCs, fibroblasts, and macrophages have been demonstrated to provide the basis for ECs behavior in the angiogenic niche during murine skin repair (26). The LCs density was positively correlated with disease activity in patients (27). Moreover, DCs maturation affects the synovial microenvironment in RA (13, 17, 28). Collectively, these results suggest that DCs are the primary initiators of inflammation-associated angiogenesis and that different DCs subtypes are essential for RA development.

### 2.2 Innate immune cells-myeloid-derived suppressor cells

**Figure 2:**
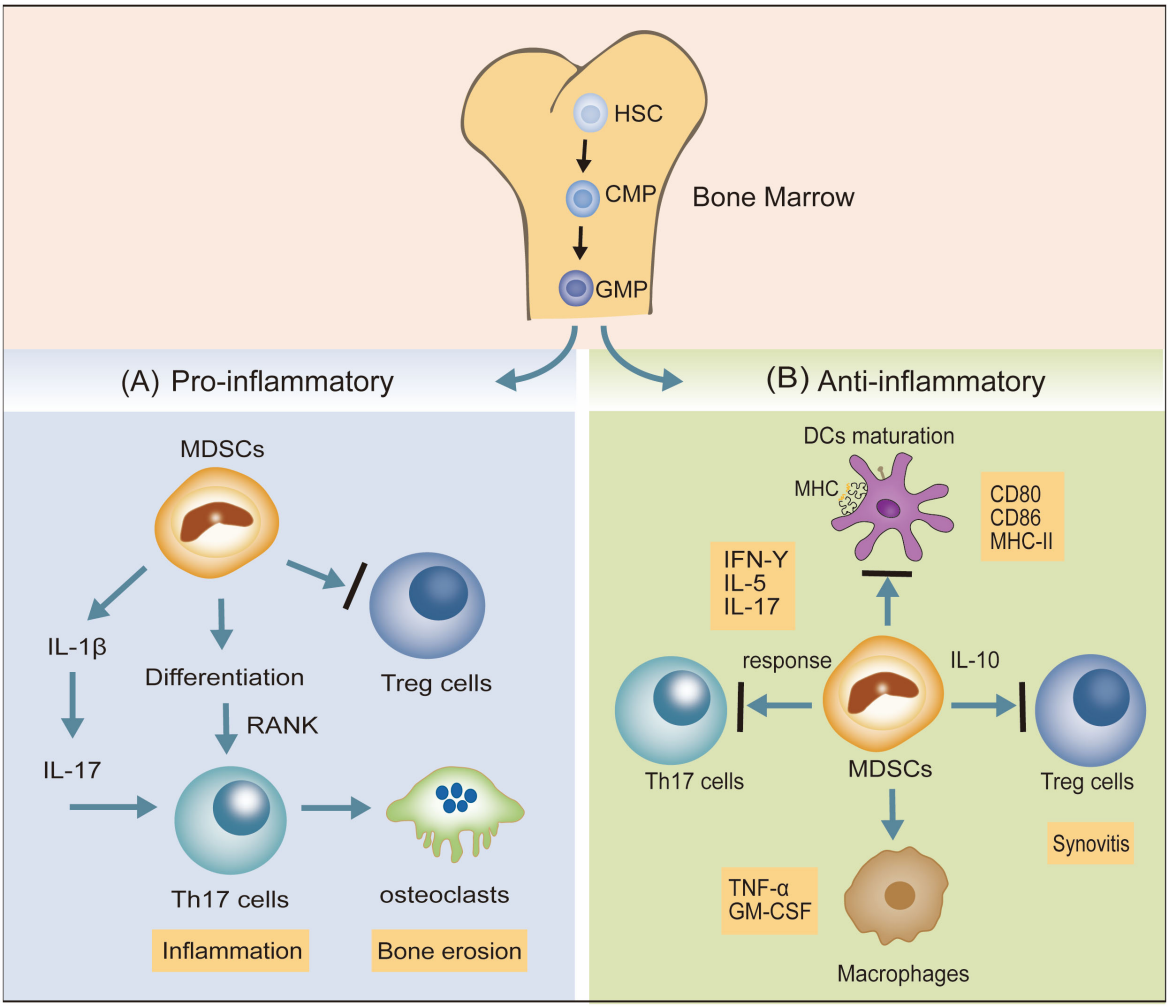
The dual inflammatory actions (pro- and anti-) of MDSCs in RA. **(A)** MDSCs promote the response of Th17 cells via IL-1β signaling and also differentiate into osteoclasts by interacting with Th17 cells and upregulating nuclear factor κβ ligand-RANK signaling, potentiating the inflammatory response. MDSCs may suppress Treg cell responses. **(B)** MDSCs inhibit the expression of DCs maturation biomarkers (CD80, CD86, MHC-II), suppress Th17 cell responses, and reduce the pro-inflammatory factors (TGF-α, GM-CSF) secreted by macrophages. MDSCs also potentiate Treg cells to depress the progress of synovitis.

Hematopoietic stem cells (HSCs) differentiate into granulocyte-monocyte progenitor cells (GMPs) via common myeloid progenitor cells (CMPs). Disruption of the differentiation of myeloid ancestors in chronic inflammatory conditions (autoimmune diseases, chronic infections, and cancer) contributes to the development of immature myeloid cells, termed myeloid-derived suppressor cells (MDSCs), which belong to a heterogeneous cell population (29). MDSCs represent a state of pathological activation of neutrophils and monocytes with substantial immunosuppressive activity (30). Dilated MDSCs exert both pro- and anti-inflammatory effects in animal models (31). The confirmed pro-inflammatory effects of MDSCs include the following: (1) MDSCs promote the response of Th17 cells by IL-1β signaling, potentiating the inflammatory response (32). (2) MDSCs also differentiate into osteoclasts via interaction with Th17 cells and upregulation of nuclear factor κβ ligand-RANK signaling, enhancing bone erosion and synovitis (33). (3) MDSCs suppress the immune-suppressing Treg cell response. MDSCs exhibit the following anti-inflammatory effects: (1) MDSCs have an inhibitory effect on DCs maturation. (2) MDSCs depress the proliferation of autoreactive T cells and reduce Th17 cell responses. (3) MDSCs may potentially reduce macrophage numbers, leading to a reduction in pro-inflammatory factors secreted by macrophages, namely TNF-α and GM-CSF (31). (4) MDSCs potentiate Tregs with anti-inflammatory effects (**Figure 2**).

MDSCs contribute to angiogenesis and tumor growth by secreting cytokines and enzyme, including vascular endothelial growth factor (VEGF), bFGF and MMP9 (34). It has been corroborated that CXCR2, a pivotal chemokine in MDSC recruitment, is expressed by MDSCs and is involved in endometriotic growth and angiogenesis (35). A2B receptor stimulation induces VEGF release, driving immune suppression by promoting the expansion of MDSCs, thereby accelerating tumor angiogenesis and worsening the impaired immune system (36, 37). In the course of bone defect reconstruction and its associated angiogenesis, MDSCs can augment the proliferation of ECs and increase vascular thickness and capillary density (38, 39). The role of MDSCs in RA-induced angiogenesis and their putative inflammatory-regulating mechanisms require further investigation.

### 2.3 Innate immune cells-macrophages

**Figure 3:**
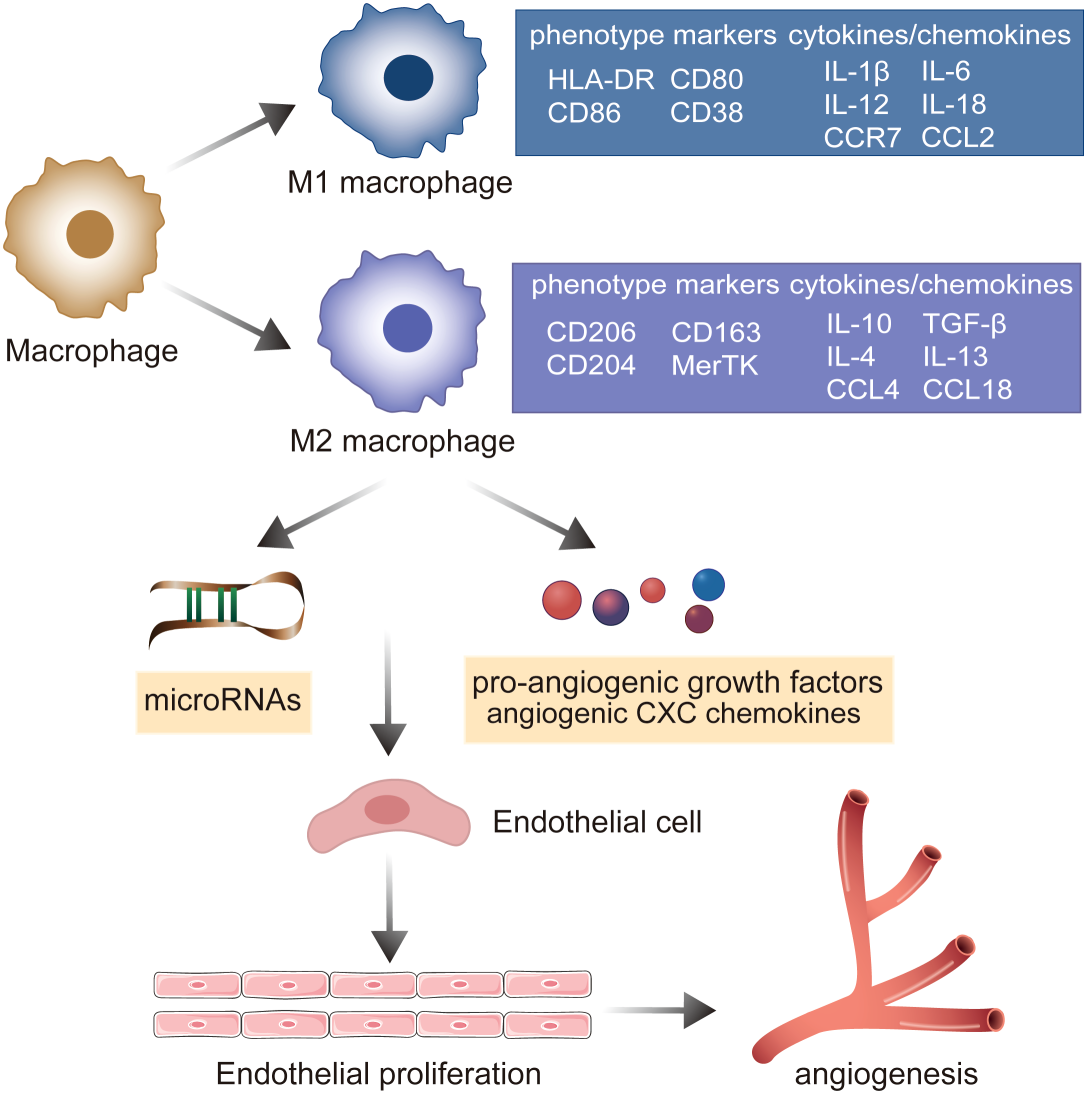
The role of macrophage polarization in the evolution of angiogenesis in RA. Under the stimulation of various pathological factors, macrophages polarize into pro-inflammatory M1 macrophages and M2 macrophages involved in the resolution of inflammation. M2 macrophages mediate microRNAs, pro-angiogenic growth factors, and angiogenic CXC chemokines to establish the cell–cell interaction with endothelium, promoting the ECs’ proliferation and potentiating the formation of the vascular network.

Macrophages are essential components of innate immunity and exhibit significant heterogeneity and polarization (40). Under the stimulation of various pathological factors, macrophages polarize to various phenotypes (mainly M1 and M2) depending on the state of and changes in the environment, and thereafter perform different functions with varying effects (41). M1 macrophages suppress sprouting angiogenesis, whereas M2 macrophages potentiate the formation of the vascular network and prolong dysregulated innate immunity by secreting a series of pro-angiogenic growth factors, angiogenic CXC chemokines, and angiogenesis-associated factors (42, 43). These induction factors augment the migration and proliferation of ECs, further activating the angiogenic switch and promoting angiogenesis (19). A recent study indicated that miRNAs play well-established roles in the interactions between M2 macrophages and ECs. M2 macrophages activate the upregulation of PCAT6 by miR-4723-5p and modulate the expression of VEGFR2, facilitating the angiogenesis of triple-negative breast cancer (44). M2 macrophage-derived exosomal miR-155-5p and miR-221-5p promote the angiogenic ability of endothelial cells (45). M2 macrophage-derived exosomes from adipose-derived stem cells trigger an exosomal pro-angiogenic effect on mouse ischemic hindlimbs by upregulating exosomal miRNA-21 delivery (46). Furthermore, M2 macrophages can deliver miR-501-3p through exosomes to elevate tube formation and repress the apoptosis of ECs through the TGF-β signaling pathway during the progression of pancreatic ductal adenocarcinoma (47). miR-199b-3p is responsible for M2 macrophage-mediated angiogenesis and immunomodulation (48). Accumulating evidence suggests that macrophages stimulated by CCL21 directly exert angiogenic effects by elevating the production of VEGF, IL-8, and Ang1 and also indirectly induce Th17 cell polarization to activate vascularization via IL-17 production in RA joints (49). IL-18 has been demonstrated to play a role in triggering macrophage M2 polarization by amplifying the expression of CD163, contributing to angiogenesis in RA (50). These findings emphasize the importance of macrophage polarization in promoting pathologic angiogenesis through cell–cell interactions with the endothelium and regulation of chemokines and cytokines in RA (**Figure 3**).

### 2.4 Adaptive immune cells

The angiogenic response in RA is orchestrated by CD4^+^ T cell subsets, especially Th17 and regulatory T (Treg) cells (51). Th17 cells, as classical proinflammatory mediators, induce the production of immune inflammatory cells and cytokines, which favor the induction of autoimmune-derived chronic inflammation and joint destruction (52). Stimulated by PlGF, an angiogenic factor homologous to VEGF, the differentiation of Th17 cells has been demonstrated to be significant in linking CD4^+^ T cells and endothelial cells. CD4^+^ T cell-derived PlGF promotes the production of IL-17 and favors the activation of ECs in RA (53). Moreover, the Th17/IL-17 related-signaling pathway has been shown to potentiate cell proliferation and foster the expression of pro-angiogenic molecules in osteoblasts (54). Tregs inhibit the activity of Th17 and other effector T cells to maintain their self-tolerance and immunosuppressive state (55). Treg cells that secrete anti-inflammatory cytokines suppress the proliferation of fibroblast-like synoviocytes (FLSs) and inflammatory angiogenesis in RA joints (56). Therefore, the crosstalk between CD4^+^ T cells and ECs, and the regulatory Treg/Th17 cell balance are essential for the progression of RA-induced angiogenesis.

In addition to CD4^+^ T cells, peripheral blood CD8^+^T cells from patients with active and in-remission RA exhibit a potently activated status and proinflammatory potential (57). Plasma VEGF levels and VEGF expression in CD8^+^T cells positively correlate with the levels of inflammatory factors in patients with RA (58). Studies have shown that CD8^+^T cells modulate pathological angiogenesis by promoting inflammation and chemokine recruitment in ocular neovascular diseases (59), and in type-2 diabetes, CD8^+^T cells’ plasticity regulates revascularization, ECs function, and angiogenesis (60). However, the role of CD8^+^T cells in RA angiogenesis remains poorly understood.

### 2.5 Activated ECs

The vascular endothelium is responsible for the dynamic maintenance of vascular tone, angiogenesis, permeability, and hemostasis and provides an anti-inflammatory, antioxidant, and antithrombotic interface for the body (61, 62). Central to the cardiovascular complications of rheumatoid arthritis are EC activation and endothelial dysfunction (63). Sustained and exaggerated activation of ECs promotes vascular endothelial dysfunction in the synovial joints and arteries of patients with RA, which can destroy inter-endothelial cell gaps and endothelial integrity. In addition, the release of vasoactive substances from the endothelium, including vascular endothelial growth factor (VEGF), nitric oxide (NO), and MMPs, further initiates endothelial proliferation, migration, and tube formation, leading to pathological angiogenesis (62, 64–66).

Neutrophils play an important role in every phase of RA, from disease initiation to chronic inflammation (67). Emerging evidence suggests that neutrophils are implicated in rheumatic disease-associated vascular inflammation via the regulation of endothelial barriers at the blood–vessel interface (68). NETosis, a modulated mechanism of neutrophil cell death, can be used to evaluate RA activity and the development of atherosclerosis in RA (69, 70). Supernatants of neutrophils cultured with serum from patients with RA, who had been treated with infliximab or tocilizumab, suppressed endothelial dysfunction by inhibiting prothrombotic mediators, pro-inflammatory factors, and adhesion molecules (69). Furthermore, tumor-infiltrating neutrophils contain a series of intracellular VEGF molecules that can be rapidly released upon stimulation to activate the angiogenic switch (71). Neutrophil extracellular traps, secreted by dying neutrophils, trigger nuclear factor κB-dependent endothelial angiogenesis in pulmonary hypertension (72). Neutrophils have been suggested to provide a functional link between inflammatory angiogenesis and RA.

**Figure 4:**
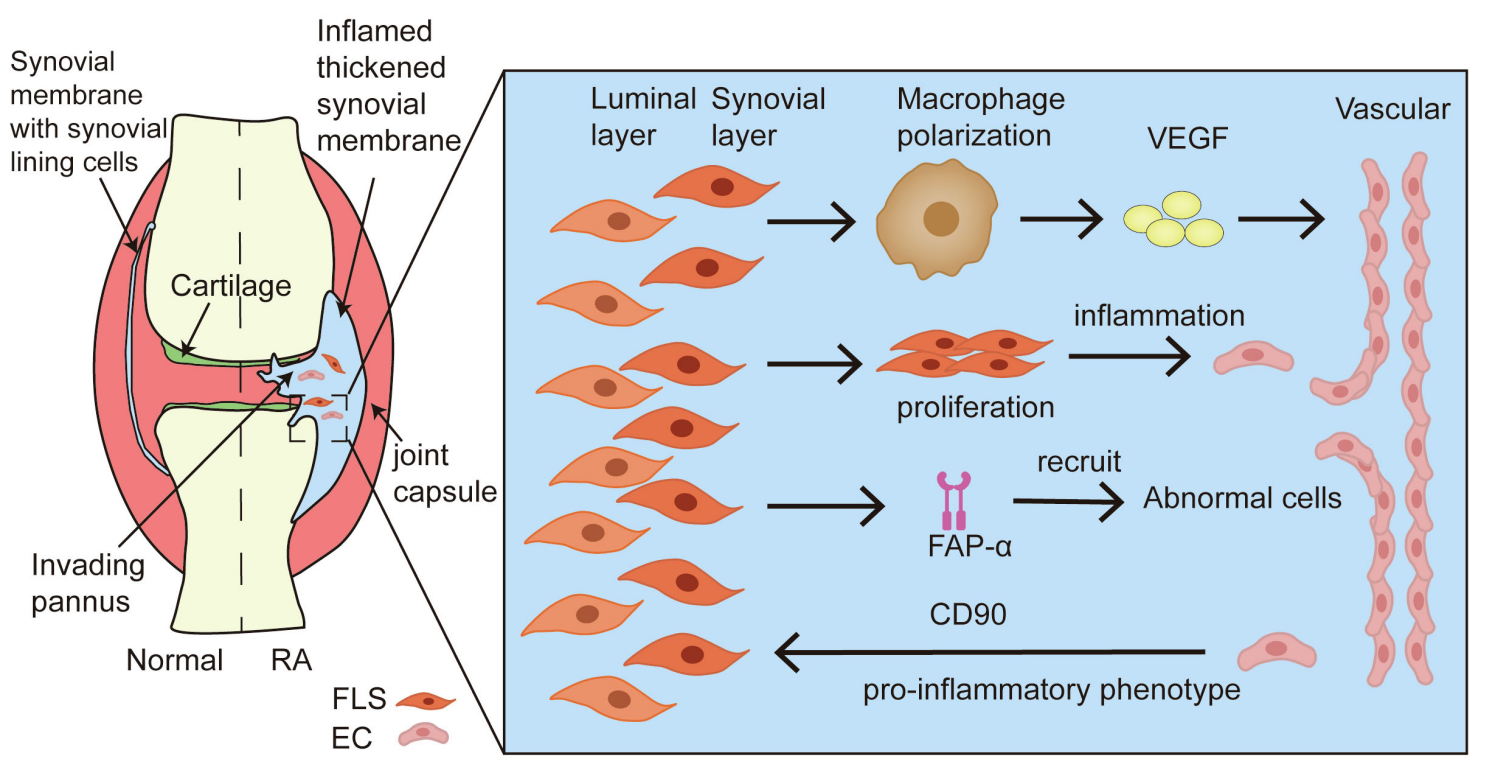
Crosstalk between FLSs and ECs in angiogenesis of RA. During the formation of pannus in the synovium tissue of RA, FLSs promote resident macrophage polarization, accelerating the secretion of pro-inflammatory mediators and pro-angiogenic factors. The aberrant proliferation of FLSs amplifies the ECs response through the activation of inflammation. FAP-α recruits and co-interacts with abnormal cells to induce local angiogenesis. In addition, ECs provide the pro-inflammatory phenotype of FLS to foster the development of synovitis.

In addition to neutrophil-EC interactions in RA models, FLSs also play a vital role. Under pathological conditions, angiogenesis and FLSs hyperproliferation synergistically trigger panel formation of pannus (73). Synovial layer FLSs polarize the interstitial macrophages and secrete pro-inflammatory mediators and pro-angiogenic factors that cause vessel sprouting (74). Furthermore, the aberrant proliferation of FLSs disrupts the dynamic balance between pro- and anti-inflammatory factors, promoting the proliferation and migration of ECs to constitute new blood vessels and amplify synovial inflammation in the RA process and joint dysfunction (75). FLSs can express a surface biomarker, namely fibroblast-activated protein-α (FAP-α), to initiate local angiogenesis by recruiting and co-interaction with abnormal cells (76). Additionally, the vasculature potently promotes the pro-inflammatory phenotype of synovial layer FLSs in the context of RA joint inflammation (77). The presence of vessels provides relative positional information for the inflammatory FLSs and enables them to respond to the maximum extent of their inflammatory potential. Inflammatory FLSs, trafficked from the intimal lining of the synovial membrane to the proximal vessel, express high levels of the pro-inflammatory marker Thy1 (CD90), which plays a large role in cartilage destruction (74). It has been confirmed that the angiogenic cytokines PlGF and VEGF secreted from synoviocytes can represent both disease activity and synovitis severity as well as the corresponding therapeutic responses to biologics in RA patients (78). Thus, the crosstalk between FLSs and ECs supports the notion that angiogenesis is essential for synovial inflammation in joints with RA (**Figure 4**).

### 2.6 Adipokines

Adipose tissue is an endocrine organ that releases a series of highly biologically active factors called adipokines, fatty acid-binding protein (FABP) 4, adiponectin, and secreted protein acidic and rich in cysteine (SPARC) (79). In the affected joints of RA patients, adipokines are implicated in both innate and adaptive immunity, increase the binding of rheumatoid arthritis synovial fibroblasts (RASFs) to EC, and are involved in the inflammatory process and cartilage reconstitution (80, 81). Recent evidence supports the link between adipokines and angiogenesis in type 2 diabetes mellitus and breast cancer (82, 83). However, the role of adipokines in angiogenesis in RA joints remains unclear.

#### 2.6.1 FABP4

FABP4 is a lipid chaperone protein belonging to the cytoplasmic FABP multigene family that regulates lipid responses and free fatty acid levels in cells (84). Several studies have demonstrated that the enhanced expression of FABP4 is correlated with RA, insulin resistance, type 2 diabetes mellitus, and cardiovascular disease (85, 86). FABP4 is expressed in the synovial lining layer by macrophages and CD8^+^ T cells, contributing to the inflammatory infiltration of the affected RA joint. FABP4 inhibitors can reduce relative inflammatory effector functions by inhibiting the secretion of IL-10 (86, 87). Furthermore, FABP4 has been found to influence *de novo* nucleotide synthesis in EC proliferation, tumor angiogenesis and growth, and asthma (88, 89). In RA patients and AIA rats, FABP4 is present in the intrasynovial adipose tissue and vessels, and it is regarded as a pivotal protein in pathologic angiogenesis and an increased risk of atherosclerotic changes (86, 90). Recent studies have shown that FABP4 is secreted by synovial M1-polarized macrophages and could be regulated by the mTORC1 pathway. BMS309403 (a FABP4 inhibitor) treatment degrades synovitis, angiogenesis, and cartilage degradation *in vivo* and *in vitro (* 91).

#### 2.6.2 Adiponectin

Adiponectin is present in macrophages and synoviocytes and modulates proinflammatory cytokine secretion during the pathophysiology of RA (92, 93). Adiponectin can exacerbate B cell proliferation and differentiation by activating the PI3K/Akt1/STAT3 axis (94). Adiponectin can also stimulate the generation of RA FLSs T follicular helper cells by regulating the secretion of the soluble factor IL-6 (95). Adiponectin triggers the expression of osteopontin, which promotes osteoclast precursor migration in synovial fibroblasts and causes osteoclastogenesis and bone erosion in collagen-induced arthritis (CIA) mice (96). However, local AdipoR1 inhibition effectively ameliorated joint inflammation and bone destruction *in vivo (* 97). In addition, elevated levels of circulating adiponectin are positively correlated not only with disease activity and synovial thickening degree in developing RA but also with higher all-cause mortality and cardiovascular mortality in RA (98–100). Some studies have shown that adiponectin can foster the production of VEGF, vascular cell adhesion molecules-1, and MMPs, including MMP-1 and MMP-13, causing RA joint inflammation and connective tissue vascularization (92). Adiponectin induces VEGF-dependent angiogenesis in synovial fibroblasts in rheumatoid arthritis via MEK/ERK signaling and miRNA-106a-5p (101). Adiponectin, secreted by synovial cells, activates cell migration, invasion, and angiogenesis, thereby promoting the pannus of arthritic joints (102). This result adds support to the notion that adiponectin plays a critical role in angiogenic activity in inflamed joints in RA.

#### 2.6.3 SPARC

SPARC is a nonstructural matricellular protein of the extracellular matrix (ECM) involved in cell-cell and cell-ECM adhesive interactions. SPARC affects immune system functions, including DCs migration, MDSCs proliferation, and functional differentiation (103, 104). Because of these properties, SPARC has been demonstrated to be associated with RA susceptibility in the Chinese Han population (105). A growing number of studies have suggested that the downregulation of SPARC is likely to be a major precipitating event in the pathogenesis of rheumatoid arthritis (103). SPARC is expressed in the synovium and synovial fluid of the affected joints of patients with RA and the CIA model. SPARC-based nanomedicines have strong potential for rheumatoid arthritis therapy by inhibiting the secretion of pro-inflammatory cytokines (106). In addition, SPARC amplified M2-polarized macrophages to activate the proliferation, migration, and angiogenesis of cholangiocarcinoma cells by modulating PI3K/AKT signaling (107). Overexpression of SPARC is capable of disrupting the integrity of vascular endothelial cell layers, affecting matrix composition and cell adhesion, thus facilitating the invasion and metastasis of tumors (106, 108, 109). SPARC knockdown reduces the expression levels of inflammatory factors and VEGFR, downregulating ECs apoptosis, and angiogenesis in diabetic retinopathy (110). SPARC is a key factor in angiogenesis and endothelial barrier function which is well established; however, the underlying mechanism of RA-induced angiogenesis has not yet been defined.

## 3 Notch signaling modulates angiogenesis in RA

### 3.1 Notch signaling

#### 3.1.1 The canonical Notch signaling pathway

**Figure 5:**
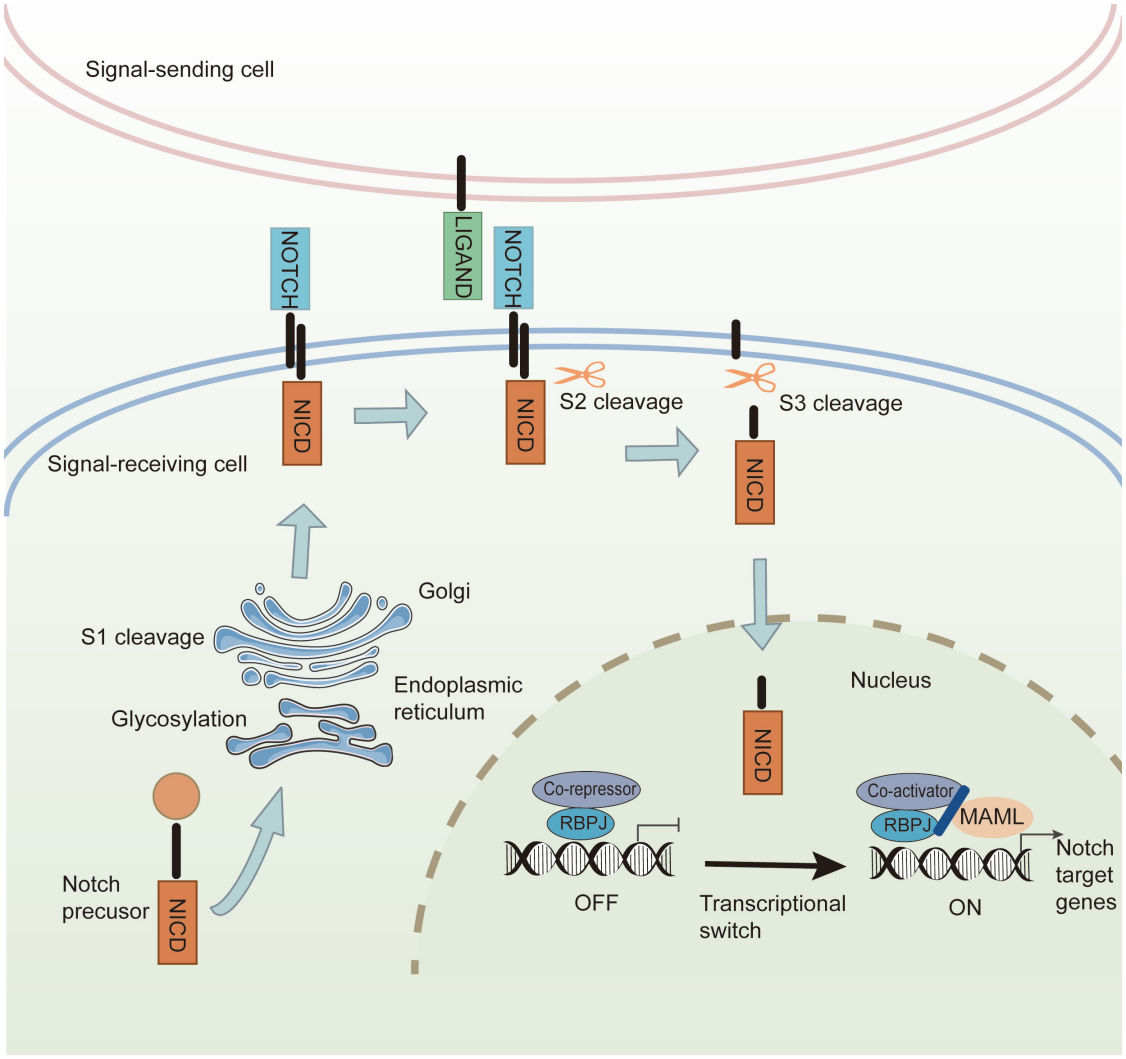
The canonical Notch signaling pathway. In the signal-receiving cell, Notch precursors are glycosylated by the endoplasmic reticulum and undergo S1 cleavage in the Golgi. The mature Notch receptors are transported into the cell membrane. Canonical Notch ligands bind to the Notch receptors, and Notch signaling induces cleavage at the S2 site. Then S3-cleavage is catalyzed by γ-secretase, causing the release of NICD. The NICD is transferred into the nucleus, where it interacts with transcriptional regulators and the coactivator to promote the transcription of Notch downstream target genes.

The Notch signaling pathway is ubiquitous in all mammalian species and participates in cell differentiation, survival, proliferation, and cell fate decisions during development and morphogenesis (111). Notch signaling comprises four parts: ligands (Delta-like 1,3,4 or Jagged-1, 2), receptors (Notch 1–4), DNA-binding proteins, and downstream target genes. Notch receptors consist of three domains: an extracellular domain (NECD), a transmembrane domain (NTM), and an intracellular domain (NICD), which are partially similar to Notch ligands (112). The structure of extracellular domains in Notch ligands and receptors contains multiple EGF-like repeats. In signal-receiving cells, Notch precursors from the endoplasmic reticulum are glycosylated at the epidermal growth factor (EGF)-like repeat domain and translocated into the Golgi apparatus. Next, mature Notch receptors are cleaved by S1 and transported to the cell membrane. Through constitutive endocytosis, ubiquitin ligases promote Notch receptor ubiquitination and degradation via the proteasome. The remainder binds to Notch ligands on the cell membrane to transmit signals (8). The canonical Notch signaling pathway includes the following steps: (1) Mature Notch receptors bind to Notch ligands, exposing the S2 site for cleavage and initiating the Notch signal. (2) The product of S2 cleavage is further cleaved in the NTM at the S3 site, and NICD as the principal effector form of Notch signaling is released. (3) NICD is transferred from the cytoplasm to the nucleus, triggering the transcription of downstream Notch target genes (113) (**Figure 5**).

#### 3.1.2 Notch signaling as a target controlling angiogenesis

Endothelial VEGF and DLL-4/Notch signaling constitute a feedback loop in angiogenesis (114–116). VEGF signaling induces the expression of the Notch ligand delta-like ligand 4 (DLL4), which activates Notch signal transduction in adjacent ECs. Notch signaling enhances VEGFR1 expression and inhibits the expression and function of VEGFR2 in neighboring cells. Thus, the level of VEGFR2 in targeted EC is higher than that in adjacent EC, which in turn increases the relative sensitivity to VEGF and endothelial proliferation (117). In contrast, DLL-4/Notch signaling is involved in a feedback loop with VEGF and confines the response of ECs, preventing excessive angiogenesis. This was confirmed by a subsequent study, which demonstrated that the levels of VEGF-A immunostaining and transcripts were significantly increased, and the expression of ESM1, a tip cell marker and VEGF-regulated gene product, was also upregulated in mice with EC-specific inactivation of the Delta-like 4 (Dll4) gene. This study further demonstrated that mice with loss of one or both VEGF-A alleles in ECs exhibited decreased levels of retinal vessel growth, branching, and EC area in the frontal region and in the central plexus. EC density and sprouting in proximity to the avascular region expressing VEGF-A were restored by the administration of an inhibitor of Notch signaling (115). Further studies are required to unravel the role of Notch signaling as an additional target to control angiogenesis.

#### 3.1.3 Notch signaling involvement in angiogenesis of RA

There is a link between Notch signaling and RA pathogenesis. Stromal cells and adipokines have been implicated in multiple studies on RA, with roles in various pathways, including inflammatory arthritis and angiogenesis. An early study found that inhibition of Notch signaling by γ-secretase inhibitors (GSI) effectively blocked Th1 and Th17 cell responses and ameliorated CIA-induced arthritis (118). A follow-up study found that GSI reduced the severity of inflammatory arthritis by regulating the expansion and function of Tregs in both CIA and collagen antibody-induced arthritis (CAIA) mice (119). In TNF-Tg mice, thapsigargin, a Notch inhibitor, suppresses M1 macrophage formation, fosters the M2 macrophage phenotype, and alleviates joint lesions (120). Notch signaling plays a vital role in controlling osteoclast differentiation and bone-resorbing activity either directly acting on osteoclast precursors, or indirectly acting on cells of the osteoblast lineage and cells of the immune system (121). In RA-affected joints, Notch1 is overexpressed and activated in FLS, Th17 cells, and M1 macrophages, accelerating the production of pro-inflammatory cytokines TNF-α, IL-6, and IL-17, leading to inflammation, bone destruction, and joint bone loss (122).

Recent studies have suggested that endothelium-derived Notch signaling modulates synovial fibroblast identity. Notch signaling increases mural cell and CD90 (THY1)^+^ sublining fibroblast differentiation and promotes the development of inflammatory arthritis (123). In synovial explant cultures, Notch signaling modulates VEGF/Ang2-induced angiogenesis, EC invasion, and pro-inflammatory cytokine secretion (124). Administration of GSI suppresses the proliferation of RA FLSs and secretion of inflammatory cytokines as well as angiogenesis, suggesting the importance and versatility of Notch signaling in angiogenesis in RA (125).

The role of FABP4 in angiogenesis has been well described. Further research underscored that Dll4-Notch signaling modulates FABP4 mRNA and protein expression in human endothelial microvascular ECs. Notch signaling, a repressor of angiogenesis, regulates fatty acid transport across the endothelium by enhancing FABP4 expression, affecting endothelial metabolic and vascular remodeling in the adult heart (126). Chromatin immunoprecipitation showed that NICD associates with FOXO1-binding sites and RBPJκ-binding motifs in the FABP4 promoter, promoting gene transcription (126). Endothelial-specific genetic ablation of Rbp-jκ elevated cardiac blood vessel density and impaired heart function, causing heart hypertrophy and failure. FABP4 upregulation following stimulation of VEGF-A/VEGFR2 and/or the Notch pathway suggests that FABP4 expression depends on Notch-DLL4 signaling (127). FABP4 secreted by M1-polarized macrophages established an essential role in synovitis, angiogenesis, and cartilage degradation in RA (91). FABP4 regulated by Notch signaling may be involved in RA angiogenesis.

In a new report of RNA-sequence analysis, SPARC expression was found to be negatively correlated with Notch signaling in 144 adenoid cystic carcinomas. Exogenous SPARC reverses Notch-induced osteoclast differentiation, bone metastasis, and bone resorption (128). SPARC-overexpressing neuroblastoma cells inhibit the expression of Notch signaling and VEGFR2 phosphorylation and suppress tumor-induced angiogenesis by limiting the formation of endothelial capillary-like tubules, the proliferation of ECs, and the generation of pro-angiogenic factors, including VEGF, FGF, PDGF, and MMP-9 (129). These findings suggest that SPARC may associate with Notch signaling and play a key role in angiogenesis.

### 3.2 Epigenetic regulation of Notch signaling

Epigenetic regulation causes heritable changes in gene expression without DNA sequence changes and includes DNA methylation, histone modifications, non-coding RNAs (ncRNAs), chromatin remodeling, and RNA modifications (130, 131). Notch signaling is mainly regulated by histone modifications and non-coding RNA in regulatory regions (132).

#### 3.2.1 The regulation of Notch signaling during angiogenesis by PTM in RA

Recent evidence indicates that post-translational modifications (PTMs) in histones are covalent modifications that play a pivotal role in controlling the dynamic fine-tuning of Notch activity (133). PTMs modulate the Notch pathway function or determine the final output of Notch signaling by regulating protein-protein interactions and establishing new binding sites. Notch ligands, receptors, and transcriptional complexes can be modified by PTMs (132). Several PTMs, including glycosylation, ubiquitination and SUMOylation, may participate in regulating Notch signaling during RA angiogenesis.

Glycosylation occurs predominantly in angiogenic regulators that modulate migration and proliferation of ECs. Notch regulation is influenced by glycosylation status, which modulates DLL4 and Jagged1 ligand responsiveness (134). According to the different connection modes of glycoprotein side chain and protein amino acid group, glycosylation is divided into N-linked and O-linked glycosylation. In the various modification types of O-linked glycosylation, O-GlcNAc glycosylation has been studied well in recent years due to the lack of fixed characteristic sequence and the complexity of structure (135). O-GlcNAc glycosylation has been demonstrated to play a critical role in activating Notch signaling, promoting Treg cell differentiation, and inhibiting T cell infiltration in autoimmune hepatitis (136, 137). O-GlcNAc glycosylation amplifies DLL4-mediated Notch signaling, which has been implicated in retinal angiogenesis and pathological vascularization (138). A higher expression of O-GlcNAcylation was exhibited in human RA synovial tissues and RASFs. Treatment of Thiamet G, an inhibitor of O-GlcNAcase, inhibited the IL-1β-induced IL-6 and IL-8 production in human RASFs *in vitro (* 139). Furthermore, dynamic changes in O-GlcNAcylation are required for osteoclastogenesis. TNF-α fosters the occurrence of O-GlcNAcylation to promote osteoclastogenesis in inflammatory arthritis. Targeted pharmaceutical or genetic inhibition or knockdown O-GlcNAcylation could ameliorate bone loss in experimental arthritis (140). The role of glycosylation of Notch signaling in RA angiogenesis is worth further exploration.

Ubiquitylation of the Notch receptor is essential for NICD degradation and Notch incorporation into the intraluminal vesicles of maturing endosomes (141). Ubiquitin-specific peptidase 10 (USP10), a deubiquitinating enzyme, interacts with NICD1 to slow the ubiquitin-dependent turnover of the activated Notch-1 receptor, thereby eliminating the ubiquitination of NICD1 and stimulating Notch signaling in ECs (142). A previous study showed that B-cell chronic lymphocytic leukemia/lymphoma6-associated zinc finger protein (BAZF) undergoes polyubiquitination in a CUL3-based E3 ligase complex, accelerating the degradation of CBF1 and abridging vascular plexus formation by mediating VEGFR and Notch cross-signaling (143). The alteration of ubiquitin proteasomal pathway causes dysregulated cellular homeostasis, progressively leading to RA. For example, polyubiquitination of TRAF2, TRAF6, RIP-1 and NEMO activated TAK1 complex, which in turn is associate with IKK activation, ultimately leading to chronic joint damage in a patient of RA (144). Methotrexate, used for the treatment of RA, inhibits the K48-linked polyubiquitination of Numb and promotes Notch-1 ubiquitination, thus restricting the expression of Notch-1 (145). Therefore, ubiquitination of Notch signaling may be related to RA angiogenesis.

Studies have shown that NICD1 is modified by SUMOylation upon stress stimulation. SUMOylation of NICD1 can amplify nuclear localization and suppress the expression of Notch target genes (146). SUMOylation of NICD1 occurs in the RBPJ-associated molecular domain to modulate Notch target gene expression at unique lysine residue sites (K1774, K1780, K1781, and K1782) in several species, including mice, rats, and humans. Stress-induced SUMOylation of NICD1 increases nuclear localization and transcriptional activity but does not influence the formation of transcriptional complexes, further regulating NICD1 nuclear translocation and stability (146, 147). Given the effect of SUMOylation on angiogenesis, deSUMOylated VEGFR2 was tested in pathological angiogenesis. SUMO-specific protease 6, a member of the sentrin-specific protease (SENP) family (SENP1, 2, 3, 5, 6, and 7), is responsible for the depolymerization of SUMO chains, deSUMOylating VEGFR2, enhancing VEGFR2 transport to the surface, and triggering the VEGF signaling cascade in AGEs to induce EC angiogenesis (148). SUMOylated VEGFR2 at the lysine K1270 site accumulates in the Golgi apparatus of ECs, reducing its surface expression and distribution and leading to retarded progression of retinal vascular angiogenesis (149). Angiogenesis is regulated by the SUMOylation of Notch-activated Dll4. SENP1-mediated Notch-1 deSUMOylation limits the cleavage of N1ICD and triggers subsequent co-transcriptional activity in the nucleus, resulting in the activation of VEGFR signaling and endothelial angiogenesis. Endothelial SENP1 deletion significantly abrogates retinal vascularization by increasing the endothelial Notch-1 response (146). Recent research showed that the expression of SUMO-1 and SUMO-2 were elevated in FLSs of RA patients. SUMO-1 proteins are mostly localized in the nuclei of synovial sublining cells (150). The role of SUMOylation of Notch signaling in RA angiogenesis is unknown.

#### 3.2.2 The regulation of Notch signaling during angiogenesis by lncRNAs/miRNAs in RA

Long nc-RNAs (lncRNAs) and short ncRNAs (such as microRNAs [miRNAs]) are a class of ncRNAs that regulate gene expression by interacting with DNA and RNA. A recent study revealed that Linc00514, a long nc-RNA, is highly expressed in clinical tissues and breast cancer cell lines. Overexpression of Linc00514 amplified M2 macrophage polarization via the Jagged1-mediated Notch signaling pathway to regulate breast cancer tumorigenicity (151). Additionally, lncRNA BREA2 disrupts WWP2-mediated ubiquitination of Notch-1 signaling to drive breast cancer metastasis (152). LINC01123 also activates Notch signaling to accelerate lung adenocarcinoma malignancy (153). In contrast, Notch signaling regulates the downstream target lncRNA LUNAR1, further triggering oncogenic activity in colorectal cancer and T-cell acute lymphoblastic leukemia (154, 155). LncRNAs involved in interactions with the Notch pathway have been linked to a variety of malignancies and tumor microenvironments (156).

The dysfunction of lncRNAs has been shown to be associated with angiogenesis through the modulation of Notch signaling. For instance, Meg3 is a tumor suppressor lncRNA that represses the expression of Notch and its target genes (Hes-1 and Hey-1) during angiogenesis after ischemic stroke (157). lncRNA Xist has been reported to be geared toward X-inactive specific transcripts and regulates the expression of Notch-1 in chronic compressive spinal cord injury-induced angiogenesis by binding to miR-32-5p (158). MALAT1 is decreased in the peripheral blood of RA patients and regulates changes in the Notch signaling pathway (159). It has been reported that lncRNA H19 inhibitors inhibit the proliferation and increase the apoptosis of synovial cells in RA by suppressing Notch signaling (160).

MiRNAs are a short functional endogenous class of RNA molecules that are 21–25 nucleotides long and are involved in most physiological and pathological events. Many studies have focused on the negative correlation between miRNAs and Notch signaling in cancer (161). However, miR-124 is a tumor-suppressive miRNA that is silenced by tumor-specific methylation during pancreatic cancer metastasis. MiR-124 recruits and activates macrophages and is transferred to a tumor-supporting M2-like phenotype by directly downregulating Notch signaling in cancer cells (162). Similarly, miR-34b-5p functions as a tumor suppressor in retinoblastoma by dysregulating the Notch signaling pathway (163). MiR-525-5p inhibits the activation of Notch and Wnt signaling pathways to reduce oxaliplatin resistance in breast cancer (164). In hepatic fibrosis, upregulation of miR-148a-5p inhibits the expression of the Notch2 and the Notch signaling pathway in human umbilical cord mesenchymal stem cells (165).

Many miRNAs interact with Notch and play pivotal roles in angiogenesis. For instance, miR-24-3p directly abrogates the expression of Notch-1 and DLL1, thereby reducing EC survival, proliferation, and angiogenic capacity in the limb muscles (166). MiR-652-3p can also limit the Notch ligand Jagged-1 to exert various effects on angiogenesis and immune cell regulation (167). MiR-223-3p has been reported to be a downstream molecule of Notch signaling that modulates the proliferation, differentiation, migration, and sprouting of ECs (168). Notch activation affects miR-223 transcription and enhances cytokine production by macrophages in patients with RA (169).

## 4 Notch signaling mediates hypoxia-induced angiogenesis in RA

Hypoxia is a key driving force inducing HIF stabilization. HIF-1α is a subunit of a heterodimeric transcription factor, and its expression is modulated by oxygen availability (170). HIF-1α is rapidly upregulated in hypoxia and degraded in normoxia. Increased HIF-1α is highly involved in the synovial tissues of patients with RA and is recognized as a crucial element in the perpetuation of angiogenesis and joint destruction in inflammatory arthritis (12). The interaction between HIF-1α and Notch signaling was elucidated in the hypoxic cellular response of cancer and RA synovial fibroblast cells (171). Activated N- and C-terminal regions of HIF-1α are involved in the hypoxia-dependent activation of Notch signaling. The HIF-1α-containing γ-secretase complex directly increases the cleavage of Notch ICD (172). The activated Notch signaling further potentiated the interaction of HIF-1α with the NICD and the CSL transcription factor, thereby increasing Notch signaling (173). Protein–protein interaction studies verified the crosstalk between HIF-1α-Notch signaling. Notch inhibitors limited the activation of HIF-1α (174, 175). Notch signaling inhibits hydroxylation of HIF-1α and recruitment of HIF-1α to corresponding sites, thereby enhancing the stability of HIF-1α (173). Recent studies revealed that under normoxia, factors inhibiting HIF hydroxylate NICD1 cause the recruitment of NICD1-deubiquitinating enzymes, consequently influencing non-degradative ubiquitin chains (176). HIF-1α-regulated miR-1275 exerts tumorigenic effects on cancer stem cell properties by modulating Notch signaling (177).

Several studies have shown that Notch signaling modulates hypoxia-induced RA angiogenesis both *in vitro* and *in vivo* (12). In RA synovial cultures, Notch-1 siRNA or antisense oligonucleotides suppress abnormal proliferation, the pro-inflammatory phenotype of FLSs, and hypoxia-induced angiogenesis (12). Notch signaling also regulates the hypoxia-induced invasion of RA-inflamed joints by influencing the VEGF/angiopoietin-2 pathway (10). The crosstalk between HIF-1α and Notch signaling has been shown to trigger the activation of synovial fibroblasts in RA. Silencing HIF-1α with small interfering RNA abridged the expression of N1ICD exposed to hypoxia in RA synovial fibroblast cells (RASFCs). Similarly, Notch-1 small interfering RNA abrogated VEGF, MMP2, and MMP9 expression in hypoxia-induced RASFCs, thus inhibiting their invasive capacity and pannus formation. However, N1ICD overexpression had the opposite effect, potentiating these processes (171).

HIF-Notch signaling has been shown to mediate angiogenesis by regulating the epigenetic modification factors in various diseases. HIF-Notch signaling has been shown to mediate angiogenesis in various diseases. Epigenetic modification factors have provided significant evidence of this.For instance, the MiR-497∼195 cluster, belonging to the miR-15 family, negatively regulated osteogenesis and angiogenesis through sustaining endothelial Notch and HIF-1α activity in murine long bones (178). In the TRIM family, TRIM28 is the most constitutively expressed transcription factor. The latest research on biological function in the cardiovascular system suggests that TRIM28 plays a pivotal role in controlling the VEGFR-Notch signaling circuit in sprouting angiogenesis by mediating HIF-1α and RBPJκ (179). In addition, HIF-1α-Notch-VEGF signaling has been reported to contribute to angiogenesis in temporomandibular joint osteoarthritis, missed abortion, and diabetic retinopathy (180–182).

## 5 Angiogenesis regulated by Notch signaling in RA: new insights into therapeutic strategies

Based on the various mechanisms mediated by Notch signaling, a suitable anti-RA approach has been developed, and promising novel strategies targeting the Notch pathway are currently under investigation (**Table 1**).

**Table 1:** Drugs targeting the Notch signaling assessed in RA.

Drugs	Model	Signaling network	Remark	Ref
**DAPT**	Mice with CIA	Notch-1	DAPT-loaded hyaluronan nanoparticles inhibited secretion of pro-inflammatory cytokines (TNF-α, IFN-γ, MCP-1, and IL-6, 12, 17) secretion and expression of collagen-specific autoantibodies (IgG1 and IgG2a).	(12)
	RAFLSs	Notch-1	DAPT inhibited TNF-α stimulated IL-6 secretion	(10)
	transgenic TNF-expressing mice	noncanonical NF-κB pathway	DAPT prevented bone loss and osteoblast inhibition and decreased osteoblast differentiation of mesenchymal stem cells.	(171)
	Mice with CIA	Notch signaling	DAPT reduced Th1- and Th17-cell responses, Th17 cell expansion from naïve T cells, and the levels of IFN-γ and IL-17.	(178)
	collagen II Cultured-Spleen mononuclear cells	Notch-3	DAPT limited the proliferation of collagen-specific T cells, and Th1- and Th17-cell responses	(179)
	RA synovial explants induced ECs	VEGF/Ang2	DAPT increased the expression of IL-6, IL-8, MMP2, and 9 and promoted EC invasion, angiogenesis, and migration	(180)
	RASFCs	Notch-1/HIF-1α	DAPT inhibited hypoxia–induced angiogenesis, EC migration, invasion, pro-MMP-2, and pro-MMP-9 activities	(181)
**LY411575**	Rats with CIA	Notch-1 and Notch-3	LY411575 reduced the ankle joint destruction and the severity of ankle joint inflammation	(182)
	Rats with CIA	Notch-1 and Notch-3	LY411575 reduced CIA severity and pro-inflammatory cytokine secretion.	(183)
**Notch-1 targeting siRNA**	Mice with CIA	Notch-1	Notch-1 targeting siRNA delivery nanoparticles retard the progression of inflammation, bone erosion, and cartilage damage by suppressing the Notch-1 signaling pathway.	(184)
**Methotrexate**	arthritic model	Notch-1	MTX/Notch-1 siRNA-loaded polymeric micelles recovered the edema in arthritic animals	(185)
**Celastrol**	Mice with CIA	NF-κB and Notch-1 pathways	Celastrol-loaded micelles inhibited the repolarization of macrophages toward the pro-inflammatory M1 pheno-type via regulating the NF-κB and Notch-1 pathways.	(118)
**Nimbolide**	Freund’s adjuvant-induced inflammatory arthritis	STAT-3/NF-κB/Notch-1 signaling	Nimbolide suppressed oxidative stress, levels of pro-inflammatory cytokines, and upregulation of MAPK, STAT-3, and NF-κB phosphorylation mediated Notch-1 signaling pathway.	(186)
**Qi-Sai-Er-Sang-Dang-Song Decoction (QSD)**	human fibroblast-like synoviocytes (HFLSs)	Notch-1/NF-κB/NLRP3 pathway	QSD inhibited inflammation in TNF-α-induced HFLSss via regulating Notch-1/NF-κB/NLRP3 pathway	(124)

The biological effects of Notch signaling are largely dependent on γ-secretase activity. Numerous studies have demonstrated that DAPT, an oral pharmacological inhibitor of active γ-secretase, attenuates arthritic symptoms and joint damage in CIA-treated and transgenic TNF-expressing mice (183–185). DAPT suppresses T cell proliferation and mediates Th1- and Th17-cell responses in RA both *in vivo* and *in vitro* (118, 186). The regulatory effect of Notch signaling on angiogenesis has been well established in several studies. DAPT inhibits EC invasion, migration, and hypoxia-induced angiogenesis in RASFCs (12, 124). LY411575 is another-secretase inhibitor that suppresses the expression of N1ICD and N3ICD, which can improve ankle joint destruction and reduce the production of pro-inflammatory cytokines (171, 187). Similarly, Notch-1 transfected by siRNA inhibited hypoxia-induced RASF invasion, angiogenesis and epithelial-mesenchymal transition *in vitro*, whereas overexpression of Notch-1 promoted these processes (188). Notch-1 targeting siRNA delivery nanoparticles inhibited inflammation, bone erosion, and cartilage damage in a CIA model, indicating that targeting Notch signaling could potentially prevent or delay the onset of RA in inflamed joints.

Methotrexate is a first- or second-line disease-modifying anti-rheumatic drug used to treat RA effectively. Nanomedicine-based MTX and Notch 1 siRNA delivery have been demonstrated to suppress paw thickness and disease progression by downregulating Notch-1 expression in an arthritic model (189). Some Chinese herbs and natural compounds have provided new sources for the development and treatment of RA. Celastrol is an active component of Tripterygium wilfordii. Celastrol-loaded polymeric micelles abrogate the repolarization of macrophages, swelling of joints, synovial inflammation, cartilage degradation, and bone erosion by modulating NF-κB and Notch signaling (190). Nimbolide, a natural chemical constituent of Azadirachta indica, suppresses oxidative stress and inflammation by inhibiting STAT-3/NF-κB/Notch-1 signaling in both IL-1β stimulated synovial fibroblasts and a Freund’s adjuvant-induced inflammatory arthritis model (191). Furthermore, in the Tibetan Hospital of the Tibet Autonomous Region, a classical herbal formula called Qi-Sai-Er-Sang-Dang-Song Decoction (QSD) is a standard medical prescription for treating RA. QSD alleviates inflammation in TNF-α-induced HFLSss by depressing the Notch-1/NF-κB/NLRP3 pathway (192).

## 6 Conclusions and future directions

Notch signaling is an important and complex player in many aspects of angiogenesis. The modulation of Notch signaling in the angiogenic processes of RA relies on different mechanisms involving stromal cells and adipokines. Although the available data suggest that DCs, MDSCs, M2 macrophages, CD4^+^ and CD8^+^ T cells, and, in part, adipokines can activate ECs and promote ECs proliferation and migration, further studies are necessary to verify the effects of Notch signaling regulated by glycosylation, ubiquitination, SUMOylation, and lncRNAs/miRNAs on RA angiogenesis. Notch signaling is an important factor in hypoxia-induced angiogenesis in RA, and targeting Notch signaling may represent an efficient therapy for angiogenesis in RA.

## Author contributions

FZ: Writing – original draft, Conceptualization, Project administration; KA, LL and XC: Writing – review & editing, Conceptualization, Project administration; ZZ and JH: Writing – review & editing, Project administration; YH and HH: Writing – review & editing, Formal Analysis, Methodology.
